# Political Hearts of Darkness: The Dark Triad as Predictors of Political Orientations and Interest in Politics

**DOI:** 10.3390/bs11120169

**Published:** 2021-12-08

**Authors:** Edward Bell, Christopher Marcin Kowalski, Philip Anthony Vernon, Julie Aitken Schermer

**Affiliations:** 1 School of Behavioural and Social Sciences, Brescia University College at Western University, 1285 Western Road, London, ON N6G 1H2, Canada; eabell@uwo.ca; 2 Department of Psychology, The University of Western Ontario, London, ON N6A 3K7, Canada; ckowals@uwo.ca; 3 Faculty of Arts, Mount Royal University, Calgary, AB T3E 6K6, Canada; pvernon@mtroyal.ca; 4 Psychology and Management and Organizational Studies, Faculty of Social Science, The University of Western Ontario, London, ON N6A 5C2, Canada

**Keywords:** Dark Triad, Big Five, personality, politics, political orientations, liberal, conservative

## Abstract

Background: This study investigated the relationships between the Dark Triad of personality (sub-clinical psychopathy, Machiavellianism, and narcissism) and four political variables: socio-religious conservatism, support for greater economic equality, overall liberal–conservative orientation, and interest in politics. A theoretical approach that focused on the influence of the Dark Triad in large groups was provided to interpret those relationships. Methodological issues found in previous research that related to the use of abbreviated scales to measure the dark traits and the use of unidimensional indicators of political orientations were addressed. Methods: A hierarchical regression analysis was conducted to determine whether any of the three dark traits could explain variance in the aforementioned political attributes over and above that accounted for by the Big Five, sex, age, and nationality, using the full personality scales and measures of political orientation that captured both social and economic liberalism–conservatism. Results: Machiavellianism uniquely predicted lower levels of socio-religious conservatism, and both Machiavellianism and narcissism uniquely predicted lower levels of overall conservatism. Conclusions: There were important links between the Dark Triad and politics.

## 1. Introduction

Although a great deal of research has been devoted to the association between the Big Five personality factors and various political attitudes and behaviors, a growing number of empirical studies are exploring possible links between the Dark Triad and political phenomena. The Dark Triad consists of three distinct but interrelated personality traits, namely sub-clinical psychopathy, Machiavellianism, and narcissism, all of which are considered to be malevolent and socially toxic [1]. Here, we examine the association between the three dark traits and two important political characteristics: liberal–conservative political orientations and interest in politics. Liberal–conservative orientations are a foundational aspect of political studies and have been widely researched in the area of personality and politics, and they are a common theme in research on the Dark Triad and politics. Interest in politics and political engagement more broadly are another primary focus in political research, although they have received less attention in studies of the Dark Triad. The latter is unfortunate, since the issue of whether the dark traits are associated with particular political orientations could be more fully understood in light of information on whether those traits are linked to a greater or lesser interest in politics.

### 1.1. Purpose of the Study

A key goal of this paper is to bring our theoretical perspectives to bear on the issue of the Dark Triad’s association with political orientations and interest in politics. Elaborated below, these perspectives developed out of empirical findings we had made, but which, at least initially, made little sense to us. What follows is an illustration of what our perspectives have to offer by way of explaining the Dark Triad’s association with political phenomena rather than formal hypothesis testing as such.

The second purpose of this study is to address some methodological issues found in the literature on the Dark Triad and politics. As outlined below, previous research has produced inconsistent findings with regard to the Dark Triad’s possible relationships with political orientations. One possible reason for the mixed findings is that many studies do not use the full-length scales developed to measure each dark trait or to measure possible personality covariates, such as the Big Five, but instead use various shorter versions of the established scales. This is a matter of some concern, given that several studies have discussed the possible pitfalls associated with using abbreviated personality scales.

Miller et al. [2], for example, suggest that the Dirty Dozen inventory [3], which is a short-form measure of the Dark Triad, does not capture important aspects of psychopathy. Maples et al. [4], although they describe the convergent and discriminant validity of the Dirty Dozen and a second abbreviated measure, the Short Dark Triad (SD3; [5]), as “adequate” (p. 326), they recommend that the full scales be used where possible. Similarly, Bakker and Lelkes [6] report that using longer scales to measure the Big Five results in higher and more consistent correlations with political orientations, and that decreasing the number of items in a Big Five inventory tends to weaken the correlations, regardless of whether political orientations are measured unidimensionally or multidimensionally. To address the problems associated with the use of abbreviated measures of the Dark Triad and the Big Five, the present research uses the full-length scales for all personality measures. 

A second, related methodological issue to be taken into consideration arises from the fact that many studies of the Dark Triad and politics do not control for the effects of the Big Five personality traits [7], despite numerous studies showing substantial correlations between those traits and many important political attitudes and behaviors (e.g., [8,9,10,11,12]). In this study, controls for the Big Five are included in the analyses.

A third methodological issue to contend with is that many of these studies use measures of political orientations that do not distinguish between their *social* and *economic* variants, despite research outlining the advantages of doing so (e.g., [13,14,15]). To address this matter, this study uses separate measures of social and economic political orientations, as well as a global, unidimensional measure for purposes of comparison.

The next section provides a brief description of the dark traits, which is followed by a presentation of our theoretical perspectives and a review of the empirical literature.

### 1.2. The Dark Constructs and Their Emergence

Psychopathy appeared as an area of study in psychiatry and forensic psychology in the mid-twentieth century (e.g., [16]). A sub-clinical psychopathic personality is characterized by emotional coldness, a relative lack of anxiety, low empathy, thrill-seeking behavior, remorselessness, and high impulsivity [17]. Machiavellianism, derived from interpretations of the sixteenth-century works of Florentine Niccoló Machiavelli, is typified by insouciance and duplicity, a cynical view of human nature, and manipulativeness in the pursuit of personal gain [18,19]. These proclivities are accompanied by a rejection of conventional morality, which is seen as naïve or even harmful to one’s self-interest [20]. Narcissism was introduced into psychology in 1898 by Havelock Ellis [21], where it soon became an important component of Freud’s theoretical analyses and behavioral descriptions. Derived from the Greek mythological figure who fell in love with his own reflection, narcissism typically includes a sense of grandiosity, pride, entitlement, dominance, and superiority [17]. An interest in narcissism grew as the counterculture emerged in the 1960s and 1970s, with the latter being declared the “me” decade by Wolfe [22,23]. Some researchers distinguish between “grandiose” or “overt” narcissism, which is characterized by uninhibited arrogance, extraversion, and self-confidence, and “vulnerable” or “covert” narcissism, in which inner feelings of superiority and inflated self-importance are masked by anxiety, neuroticism, and hypersensitivity [24,25]. 

The three components of the Dark Triad are usually moderately and positively inter-correlated, with all three associated with low Agreeableness and low Honesty-Humility [26,27,28]. However, the three traits have consistently been shown to correlate differentially with other psychological variables [27,29,30], which suggests that they are distinct personality traits. Men tend to score significantly higher than women on all three [26], and all three are partly heritable [28,31,32].

## 2. Theoretical Perspectives

### 2.1. Political Orientations

There is not a large body of theoretical work on the Dark Triad’s association with political phenomena. With regard to the Dark Triad in general, much of the theoretically oriented research that has been done thus far tends to view the Triad as three evolved strategies that individuals may use to deal with the adaptive problems they confront in their environments [33,34,35]. One promising approach using this theme is derived from the life history theory [36,37,38], which maintains that when resources are only intermittently available or other aspects of the environment make survival precarious and unpredictable, various species adapt by developing a fast life strategy that is characterized by behavioral patterns such as sexual reproduction at a young age, having a large number of offspring, minimal parental investment in the rearing of the young, and short life spans. Figueredo et al. [39,40] suggest that Machiavellianism is part of a life strategy that is faster than the species average and report a negative association between Machiavellianism and the K-factor, which captures slow life strategy. Both environmental and genetic influences are said to play a role in the adoption of a particular life history strategy. 

Similarly, Jonason, Koenig, and Tost [41] observed that participants scoring higher on the Dark Triad tended to have faster life history strategies and were more likely to discount the future. In another study, high scores on the Dark Triad, especially psychopathy, were associated with fast life strategies, such as short-term mating schemes [42], and short-term mating has also been linked with narcissism [43,44]. The theme of the dark traits as individual adaptations to environmental challenges has also been developed by Mealey [45], who interprets psychopathy as a trait that is subject to frequency-dependent selection, in that it can thrive where a cooperative strategy predominates. 

The life history theorists mentioned above make a valid point in claiming that the dark qualities can provide fitness advantages to the people possessing them, at least in the short run or under certain conditions. Being Machiavellian, for example, is one way to obtain what one wants, one way an individual can gain advantage in the competition for survival and reproduction. Being dismissive of conventional morality—a key aspect of Machiavellianism—does reduce restrictions on what people can do to achieve their goals. Put in the language of game theory (see [46,47]), the dark traits may reflect a psychological predisposition to “defect,” which involves seeking the maximum reward for oneself rather than cooperating with others to achieve an outcome that is optimal for all the people with whom one is interacting. Given that, as mentioned, there is evidence that the Dark Triad is partially heritable, it follows that for people with high levels of these traits, such defection is to a significant degree dispositional or natural. 

A key finding in the research on evolutionary game theory is that cooperative strategies often predominate in conditions characterized by iterated interactions in which defectors may be safely ignored or can be penalized by cooperators, so one could also hypothesize that, in such situations, people with high levels of the dark traits may enjoy temporary gains but do not fare well in the long run unless they convert to cooperation [47]. Similarly, high Machiavellianism scorers have been known to act altruistically when they were being observed by others, but selfishly when their actions could not be detected, which suggests that there is a good deal of flexibility in the behavior of people exhibiting this trait [48]. 

Several researchers have sought to identify the conditions under which defecting or acting on dark impulses provides fitness advantages or disadvantages and what the trade-offs might be (e.g., [29,35]). With regard to advantages, life history theorists point out that a fast life strategy may be an efficient way of dealing with limited time horizons [34]; more generally, one could say that it may be the best evolutionary strategy under harsh conditions. Jonason et al. [35] even suggest that having high levels of the dark traits and exhibiting their associated behaviors can be glamorous or alluring. 

While these studies and their theoretical underpinnings are important, their relevance to political phenomena seem tangential at best. For one thing, they are couched in terms of the advantages or disadvantages experienced by a person having a dark trait and how such individuals may exploit other *individuals*, whereas much of what is normally conceived of as politics is concerned with how best to attend to the needs of *large groups*, such as one’s society or country. We maintain that to understand how the prevalence of the Dark Triad among the public at large may contribute to politics, which is the purpose of this paper, it is necessary to take into consideration not only the outcomes for individuals, but also the consequences for large groups.

The well-being and smooth functioning of large groups requires, among other things, cooperation and coordination among their members in order for the group to survive and compete with other groups, and a great deal has been written about how cooperation and coordination can emerge in such groups (e.g., [49,50,51]). One way to make theoretical sense of the Dark Triad’s relevance to mass politics is to view it as a manifestation of low levels of an evolved tendency to, under certain conditions, embrace in-group altruism and cooperation. This position maintains that the dark traits are the obverse, the polar opposite of the prosocial traits. To invert a term used by Nowak and Highfield [46], we see people with high levels of the dark characteristics as *super non-cooperators*. If we consider a psychological continuum with individuals having the highest levels of the prosocial traits at one end and those with the lowest levels at the other, we hypothesize that people possessing the dark traits will congregate at the lower end of the spectrum. This conceptualization suggests that the Dark Triad represents a form of *reverse altruism*. Under this theoretical approach, the dark traits are personality characteristics that interfere with the cooperation and coordination that is needed for optimal group functioning and, ultimately, for group survival. 

Some researchers claim that historically, the development of universalistic religions has contributed to greater levels of within-group cooperation (e.g., [51,52]). These are religions, such as Buddhism, Christianity, and Islam, in which membership is not restricted to people of a particular ethnicity, language, or ancestry, and for that reason can foster unity and cooperation in cosmopolitan societies. The latter two religions feature a deity that punishes bad behavior (especially when perpetrated against in-group members), which can promote certain kinds of in-group cooperation, such as abstaining from violence, helping the poor, putting others ahead of oneself, etc. The notion of karma may play a similar role in Buddhism. If, as is hypothesized here, people with high levels of the Dark Triad are super non-cooperators, it follows that they would be less inclined to strongly embrace or even gravitate toward religious group membership, which normally involves following a system of morality and behavioral imperatives imposed by others. In terms of the variables examined in this study, this theoretical position would be consistent with high levels of the Dark Traits being negatively correlated with social conservatism, since social conservatism is strongly related to religiosity [53]. This same theoretical logic maintains that the dark traits should be negatively related to economic liberalism because acting on that political orientation can be viewed as a form of cooperation or even altruism, in that it supports a redistribution of resources to needful fellow citizens. Finally, since social conservatism and economic conservatism tend to be positively (though far from perfectly) correlated [6]—people who are socially conservative are typically economically conservative as well—this perspective would maintain that the correlation between the Dark Triad and overall political orientations, which combine social and economic elements, will be lower than what is observed for either social conservatism or economic liberalism measured separately. The reason for this is that this theoretical perspective maintains that the Dark Triad is conducive to social liberalism, but also to economic conservatism. 

### 2.2. Interest in Politics

With regard to interest in politics, our theoretical position is taken from Paulhus’ [54] “spheres of control” model as applied to Machiavellianism. This model maintains that one may perceive the locus of control of events differently depending on whether the events pertain to: (a) individual goals that do not directly involve other people; (b) interpersonal matters, such as dyadic relationships; or (c) sociopolitical issues affecting society at large. Paulhus [54] provides evidence that Machiavellians tend to believe that they have a high level of control over outcomes that follow from small-group interactions, but perceive themselves as having very little control over sociopolitical matters. If this is true, and if this proclivity generalizes to the other dark traits, the locus of control perspective is consistent with the Dark Triad being negatively associated with interest in politics, the hypothesis being that people with high levels of these traits prefer to focus their attention on matters they can control and thereby maximize their chances of gaining personal advantage. 

## 3. Associations between the Dark Triad and Political Variables Observed in Previous Studies

As noted above, the findings for the Dark Triad and political orientations have been mixed. Christie and Geis [18], in their pathbreaking psychological research on Machiavellianism, concluded that the trait was not related to political outlooks. The measures used for Machiavellianism were the full Mach IV and Mach V [18], which were administered to American college students. Political orientation was determined by respondent ratings of 1964 presidential candidates Johnson and Goldwater. Similarly, Hodson, Hogg, and MacInnis [55], after controlling for the Big Five using the 44-item Big Five Inventory [56] found no significant association between Machiavellianism measured by the full Mach IV and a three-item composite measure of general, social and economic conservatism in a study of Canadian university students. Hart, Richardson, and Tortoriello [1] also observed no significant association between Machiavellianism and political ideology among an MTurk sample who completed the Short Dark Triad (SD3; 27 items; [5]) and a single seven-point political ideology self-placement scale. Jonason [57] found no significant association between Machiavellianism and political orientations, after controlling for the Big Five using the Big Five Inventory (44 items; [58]), in a Texas student sample that was given the Dark Triad Dirty Dozen (12 items; [3]) and a five-point ideology scale. Jonason [57] also observed no significant association in an MTurk sample between Machiavellianism measured using the SD3 and a composite measure of conservatism that combined indicators of political, financial, and social conservatism, after controlling for HEXACO personality traits using the 60-item HEXACO-PI-R [59]. However, in the same study, Jonason [57] did find a negative association between Machiavellianism and a composite measure of liberalism that combined indicators of political, financial, and social liberalism. Using separate measures for social and economic liberalism–conservatism, Bardeen and Michel [13] found that a grouping of high social liberalism and high economic conservatism showed the highest levels of Machiavellianism as measured by the SD3, after controlling for the Big Five using the Big Five Inventory-10 (10 items; [60]). 

Hodson et al. [55], in their previously mentioned study, found no association between narcissism measured with a reduced (20-item) version of the Narcissistic Personality Inventory (NPI; [21]) and their composite measure of general, social, and economic conservatism. In the aforementioned study by Jonason [57], narcissism measured with the Dirty Dozen was associated with conservatism as indicated by a five-point self-placement scale, although when measured with the SD3, narcissism was unrelated to separate measures of liberalism and conservatism. In a nationally representative American sample, Hatemi and Fazekas [61] found no significant relationship between the full (40-item) NPI and political orientations as indicated by a five-point liberal–conservative self-placement scale and individual items on public spending, gun control, policing, and refugees, although a significant association was found for an item on immigration, such that more narcissistic individuals had more conservative views on that topic. Hatemi and Fazekas [61] did report that the narcissism Entitlement facet was significantly associated with conservative orientations on all of these measures, while the Exhibitionism facet was significantly associated with a liberal position on all of them, except the items on immigration and policing. In the Hart et al. [1] study reviewed above, vulnerable narcissism measured with the Hypersensitive Narcissism Scale [62] was related to higher liberalism, although the latter researchers found no significant association between liberalism and grandiose narcissism using the SD3. In the Bardeen and Michel [13] research cited above, a combination of high social conservatism and high economic liberalism was associated with the highest levels of narcissism.

With regard to psychopathy and political orientations, Hodson et al. [55] in the research cited above used the full Self-Report Psychopathy-III (SRP-III) scale [63] and found no significant association between overall psychopathy and their measure of conservatism, and no significant association between conservatism and three of four psychopathy subscales. Jonason [57], in the research described earlier, found a positive association between psychopathy measured with the Dirty Dozen and liberalism in the Texas student sample, but a positive association between psychopathy measured with the SD3 and conservatism in the MTurk sample. Lilienfeld et al. [64], using the Psychopathic Personality Inventory-Revised-Short Form (PPI-R-SF; 56 items; [65]) and a single-item, five-point liberalism–conservatism scale with an international internet sample, found a positive association between overall psychopathy and conservatism, as well as positive associations between conservatism and facets of Fearless Dominance, Self-Centered Impulsivity, and Coldheartedness. Preston and Anestis [66], who used the Triarchic Psychopathy Measure [67] with a MTurk sample to examine whether the subscale traits of Boldness, Meanness, and Disinhibition were associated with 32 specific political issues, found that the three subscales tended to correlate with conservative issue orientations, although there were some exceptions. Gay et al. [14] administered the Psychopathic Personality Inventory-Revised-Short Form (PPI-R-SF; [68] to two American MTurk samples along with a single-item, seven-point liberalism–conservatism scale and found a negative association between liberalism and the Coldheartedness facet, although the association was no longer significant when controlling for the Moral Foundations Theory traits of Harm and Fairness.

The association between the dark traits and interest in politics was examined by Chen, Pruysers, and Blais [69], who reported that narcissism, as measured by the SD3, was positively associated with interest in politics, although this finding was rendered nonsignificant after controlling for the HEXACO personality traits. Fazekas and Hatemi [70], while not treating interest in politics per se as an outcome variable, found general narcissism and some of its facets, as measured with the NPI, to be related to various forms of political participation.

## 4. Method Used in the Present Study

### 4.1. Participants

The sample comprised 530 volunteer participants (444 females, 84 males, and 2 with missing gender information) who were taken from a registry created for an ongoing series of twin and sibling studies on a variety of topics. People were recruited to the registry over a number of years through a variety of different methods. Twin organizations were contacted and asked to assist with recruitment, twins were recruited from other registries after permission was granted from registry administrators, and twins were approached at the annual Twins Days festival in Twinsburg, Ohio. The data analyzed here were gathered in 2007, before the present study was conceived, with more than half the sample made up of participants whose co-twin did not take part in the study. Findings from the combination of participants and variables utilized here have not been previously published, although some of the scales have been used in other studies. The age of the participants ranged from 16 to 92 years, with an average age of 40.49 years (*SD* = 16.58). Of the total, 360 individuals were Canadian and 170 American. 

### 4.2. Materials—Measures of Variables

There is a longstanding, ongoing debate among social researchers as to whether political orientations are best described as falling along a single, left–right or liberal–conservative dimension or, alternatively, that multiple dimensions should be recognized (see [71] for a review). Those taking the latter position most commonly maintain that it makes sense to distinguish between *social* and *economic* liberalism–conservatism. For theoretical reasons, the measures used here included both a unidimensional indicator of political orientations, as well as separate measures of the social and economic dimensions.

#### 4.2.1. Political Issues Questionnaire 

The political variables were taken from the Political Issues Questionnaire [72]. To gauge interest in politics, participants in the study were asked, “Generally, how interested are you in national politics?” with response categories 1 = *Not at all interested* to 7 = *Very interested*. To obtain information on overall (unidimensional) liberal–conservative political orientations, a 7-point, single-item scale was used, with a higher score indicating greater conservatism. To introduce the item to Canadian participants, the questionnaire stated, “In politics, people sometimes talk of left (liberal) and right (conservative). Where would you place yourself on the scale below?” For the Americans, the first sentence read, “In politics, people sometimes talk of liberal and conservative,” with an identical second sentence. This kind of single-item, self-placement scale is widely used in political research. A meta-analysis performed by Sibley, Osborne, and Duckitt [73] identified 73 studies that have employed this instrument, and their analysis included only those studies that used it in conjunction with the Big Five personality traits. 

Following those measures, 42 additional Likert-style political attitude items were administered. They included items from existing scales, as well as original items devised by the authors. The items administered were chosen to provide indicators of both social and economic liberalism–conservatism. The Kaiser–Meyer–Olkin value for the items was 0.83, which indicated that the correlation matrix was appropriate for factor analysis [74]. After a visual examination of the scree plot, six factors were extracted, which accounted for 44.44 per cent of the variance. Individual attitude items with high loadings on a single factor were used to construct scales. Six of the attitude items were removed from the constructed scales because they did not load clearly onto a single factor. Linear aggregates were used to generate scale scores in which higher values represented stronger attitudes on that dimension.

One of the factors that emerged was made up of eight items that pertained to *social* liberalism–conservatism (example items: “It should be possible for a woman to get a legal abortion if she wants it for any reason,” “Things would improve if more religious people became active in politics”). (A full list of the items used in this scale and in the economic liberalism–conservatism scale discussed below is provided in [72]). The internal consistency for this factor, which in this study was labelled “socio-religious conservatism,” was good (α = 0.83).

A second factor captured important aspects of *economic* liberalism–conservatism. It was made up of nine items (“Governments should do more to reduce the gap between the rich and the poor,” “If incomes were more equal, nothing would motivate people to work hard”). This factor was labelled “support for greater economic equality.” The internal consistency of the items was acceptable (α = 0.71). The emergence of these two scales as separate factors, and the analysis that follows, highlights the advantages associated with using multidimensional measures of political orientations.

In order to assess the ability of the single self-placement item to predict respondents’ positions on the scales created by factor analysis, a one-way analysis of variance was conducted that examined the differences between left/liberal, center, and right/conservative individuals, as determined by the single-item scale. The right/conservative participants had significantly higher levels of socio-religious conservatism, while the left/liberal group scored significantly higher on support for greater economic equality [72].

#### 4.2.2. Self-Report Psychopathy Scale (SRP-III-R12)

The Self-Report Psychopathy Scale measures subclinical psychopathy [75] using 62 self-reflective five-point Likert scale items ranging from 1 (*strongly disagree*) to 5 (*strongly agree*) (“Most people are wimps,” “It tortures me to see an injured animal”). The SRP-III-R12 has demonstrated high internal consistency (α = 0.79) and is the most widely validated measure of psychopathy [27].

#### 4.2.3. MACH-IV 

The MACH-IV is a self-report measure of Machiavellianism [18] composed of 20 items (“The best way to handle people is to tell them what they want to hear,” “Never tell anyone the real reason you did something unless it is useful to do so”) and is answered on a five-point Likert scale ranging from 1 (*strongly disagree*) to 5 (*strongly agree*). Previous research has shown that the MACH-IV has acceptable internal consistency (0.70 ≤ α ≤ 0.76; [76]). Moreover, Rauthmann [77] has demonstrated that the MACH-IV has strong criterion validity, as it significantly correlated with eight Machiavellian manipulation tactics, with correlation levels ranging from 0.26 to 0.65.

#### 4.2.4. Narcissistic Personality Inventory (NPI)

The Narcissistic Personality Inventory assesses individual differences in subclinical narcissism using self-reports [17]. The NPI consists of 40 ipsative self-reflective items where participants are to choose between a narcissistic and a non-narcissistic-style response (“When people compliment me I sometimes get embarrassed” versus “I know that I am good because everybody keeps telling me so”). This scale has shown to be consistent between peer and self-ratings [78] and has demonstrably high internal consistency (α = 0.84; [27]).

#### 4.2.5. NEO Personality Inventory (NEO PI-R)

Participants completed the NEO PI-R [7], which consists of 240 items and is a frequently used inventory for assessing normal adult personality on the dimensions of the Five Factor Model. Its variables and factors have sound and well-established psychometric properties. The NEO PI-R has high internal consistency, with alphas ranging from 0.86 to 0.92 for its various scales [7].

### 4.3. Procedure

Questionnaires were sent to individuals who had previously agreed to participate in the study. Once completed, individuals returned the questionnaires in a stamped and addressed envelope (which was provided in the package); participants were informed that this would imply consent. The data were gathered in two waves—one was made up of the Dark Triad and Big Five measures, the other comprised the political questionnaire. (The use of two waves was not part of a pre-conceived sampling design. As mentioned, the data were gathered before the present study was planned.) After each wave, respondents were sent $20 and were entered into a draw to win one of ten $100 prizes upon return of the completed questionnaires. Participants also received debriefing information.

## 5. Results

### 5.1. Bivariate Correlations

Table 1 reports the correlations between the four political measures and the Dark Triad, age, and sex. Table 2 shows the associations between the political variables and the Big Five personality factors, while Table 3 contains the correlations for the Big Five, the Dark Triad, age, and sex. Although generally not especially strong in magnitude, a pattern of correlations emerged in Table 1, such that in several instances, high scores on a Dark Triad trait predicted left/liberal political orientations. Specifically, all three dark traits (especially Machiavellianism) were associated with a rejection of socio-religious conservatism, and Narcissism was correlated with an overall left/liberal political outlook. With regard to interest in politics, higher Machiavellianism scores were associated with lower levels of interest in politics.

The correlations in Table 2 indicate that of the Big Five traits, Openness most consistently correlated with the political measures, with higher scores on that trait associated with an interest in politics, an overall left-wing orientation, and lower scores on the socio-religious conservatism and support for economic equality scales. In Table 3, Agreeableness was the Big Five trait that most consistently correlated with the three dark personality factors, showing substantial negative correlations with all of them. The sizable correlations observed in Table 3 suggest that controlling for the Big Five is essential when assessing the Dark Triad as a predictor of the political phenomena examined here. 

### 5.2. Congruence of the Bivariate Correlations with Results of Previous Studies

In order to assess the comparability of the sample with samples used in previous research, in particular with regard to the measurement of overall political orientations, the Big Five, and the Dark Triad, a number of comparisons were made between the results discussed above and those reported in meta-analyses. By and large, the Pearson’s *r* correlations between overall political orientations and the Big Five found in this study are consistent with what has been observed in previous research. Table 4 compares the correlations shown in the current study with those found in a 10-country, 73-study meta-analysis (*n* = 71,895) performed by Sibley et al. [73]. In each of the studies included in the meta-analysis, political orientation was assessed using a single-item self-placement measure, which is what was used as our measure of overall political orientation.

It should be noted that only 2 of the 73 studies in the Sibley et al. [73] meta-analysis used the full, 240-item NEO-PI-R to measure the Big Five. The Big-Five Inventory (BFI; 44 items) was used in 24 of the samples; 13 used the NEO Five-Factor Inventory (NEO-FFI; 60 items); 9 used the Ten-Item Personality Inventory; 2 used the Big-Five Mini-Markers (40 items), and 1 used the revised Big-Five Questionnaire (BFQ-2; 120 items). A general personality measure from the International Personality Item Pool was used in seven studies (50 items in the only published study in this category), with two more using the related Big-Five Aspects Scale (BFAS; 100 items). Six additional studies used other trait adjective ratings (number of items unspecified), and two used the HEXACO-PI-R (60 items in the one study in this category that was published). The remaining five studies used other reduced, derived, or unspecified measures of the Big Five [73]. 

Comparing the first two rows of Table 4, it is apparent that the overall pattern of correlations is rather similar for the current study and the full, 73-study meta-analysis. In each case, the highest correlation is a negative *r* observed for Openness. The Conscientiousness *r* is the next highest correlation in both cases, with a value of 0.10 in both sets of results. Coefficients for the three remaining Big Five personality factors are quite low, with each one being below ±10. 

The only sizeable difference between the present study and the full meta-analysis appears to be for Openness, where the magnitude of the correlation is higher in our study (−0.37 compared to −0.18 in the meta-analysis). However, this higher correlation appears to be a function of the measures used to assess the Big Five. As mentioned, only 2 of the 73 studies in the meta-analysis used the full NEO-PI-R to measure personality. Tellingly, those 2 studies had higher correlations between Openness and overall political orientation than what was observed for the 73 studies as a whole. The two studies were Carney, Jost, Gosling, and Potter ([79] Sample 1), and Jost et al. ([71], Study 1), which had Pearson’s *r* values of −0.42 and −0.39 for Openness, respectively [73]. In fact, those studies showed the highest correlations for Openness out of all 73 studies included in the meta-analysis. Therefore, when controlling for the personality inventory used, the results for Openness in the present research (*r* = −0.37) match those of other studies quite closely.

Looking at the third row of Table 4, it can be seen that the five Canadian samples in the meta-analysis have *r*-levels that are fairly close to those observed in the overall meta-analysis. However, like the full meta-analysis, the Canadian studies have a lower correlation for Openness (*r* = −0.19) than that observed in the present work. Nevertheless, this difference can, again, be attributed to the personality measures used—none of the five Canadian studies used the full NEO-PI-R. Two of the five used the BFI, with the remaining three employing the BFAS, Mini-markers, and the HEXACO-PI-R. The study using the latter inventory [80] showed the highest correlation between Openness and overall political orientation of the five Canadian studies in the meta-analysis, with *r* = −0.24 [73]. Overall, then, there appears to be no reason to question the appropriateness of the present sample with regard to the measurement of overall political orientation and the Big Five.

Table 5 compares the Pearson’s *r* correlations between the Big Five personality traits and the Dark Triad found in the present study with those reported in meta-analyses conducted by Muris et al. [26] and by O’Boyle, Forsyth, Banks, Story, and White [81]. Muris et al. [26] only included studies that examined all three dark traits and all the Big Five factors, whereas O’Boyle et al. [81] also included studies that did not assess all the dark and Big Five traits. To enhance comparability with the present study, the results taken from the former work shown here only pertain to studies that used the full Dark Triad measures (*n* = 3133) and do not include findings from studies that used the shorter scales. O’Boyle et al. [81] do not compartmentalize their results on the basis of the Dark Triad measures used, and neither meta-analysis presents separate results for the various measures of the Big Five. The types of sample employed was not indicated in these meta-analyses beyond the exclusion of clinical samples in the O’Boyle et al. [81] study, although Muris et al. [26] state that it is common practice in Dark Triad research to rely on highly educated student and internet samples.

Like the findings for overall political orientation and the Big Five, the results from the current research for the correlations between the Big Five and the Dark Triad appear to be largely in line with what the other samples have produced. For example, Table 5 shows that in the current study, and in the two meta-analyses, the strongest correlations are the negative *r*s found for Agreeableness, and in all three studies the magnitudes of the Agreeableness *r*s for psychopathy and Machiavellianism exceeded those for narcissism. Overall, the similarity of the findings in the three groups of samples is quite evident. Of the 15 sets of correlations shown in Table 5, only one appears to be anomalous: the correlations for neuroticism and psychopathy. Although one can only speculate as to why the correlation for these traits found in the present study (*r* = 0.32) is of higher magnitude than those in the meta-analyses, this finding may have resulted from the use of the full NEO-PI-R in the present work. However, the larger picture provided by Table 5 indicates, again, that the correlations found between the Big Five and the Dark Triad traits observed in the present study are quite comparable to those contained in the two meta-analyses. 

In summary, the similarity of the correlations found in the current study with those reported for other samples, as illustrated in Table 4 and Table 5, indicates that the sample used here aligns rather well with those employed in previous research. 

### 5.3. Hierarchical Regressions

In order to further understand how the Dark Triad and the four political characteristics of interest are related, each of the political measures were regressed hierarchically, such that the predictor variables in the first model included only sex, age, and nationality (Canadian vs. American), with the Big Five traits added in the second model and the Dark Triad added in the third. (Sex was also tested as a moderating variable, but no significant moderation was observed.) Hierarchical regression, and in particular this ordering of the variables, was chosen in order to examine the extent to which the Big Five could explain unique variance beyond that accounted for by the three demographic variables and whether any of the three dark traits remained significant predictors after placing the other two sets of variables in the model. The results are shown in Table 6. 

With regard to interest in politics, the addition of the Dark Triad did not significantly improve the overall variance explained. Of the Big Five, only Openness was significantly associated with political interest when all predictor variables were included. For overall liberal–conservative orientation, the addition of the Dark Triad did significantly improve the model fit, however modestly, with both narcissism and Machiavellianism being significantly related to a left-wing political orientation. Again, among the Big Five traits only Openness was a significant predictor in the full model. 

The Dark Triad also explained unique variance in socio-religious conservatism, primarily through a substantial beta weight for Machiavellianism (β = −0.29, *p* < 0.001). Another notable finding with regard to this outcome variable is that in model 2, which contained only the demographic variables and the Big Five, Openness and Agreeableness were highly significant predictors of this political trait, but in model 3, Agreeableness became nonsignificant with the inclusion of the Dark Triad. None of the dark traits explained unique variance in attitudes regarding support for greater economic equality.

## 6. Discussion and Suggestions for Further Research

With regard to political orientations, the theoretical position advanced here that sees people with high levels of the dark characteristics as super non-cooperators in a large group context suggests that the Dark Triad should be associated with social liberalism. The findings of this study for Machiavellianism are consistent with this perspective, in that people with high levels of that trait were more likely to reject socio-religious conservatism. However, this tendency does not appear to be applicable to the other two dark traits. 

Why would Machiavellians have an aversion to socio-religious conservatism? Research by Lee and Ashton [83] indicates that the HEXACO Honesty-Humility (HH) factor captures (at the opposite end of the spectrum) the common element shared by each of the Dark Triad traits. Silvia, Nusbaum, and Beaty [84] report findings in which HH was associated with higher levels of global religiosity, intrinsic religiosity, and fundamentalism, and lower opposition to Christianity. The latter researchers also found that the Fairness facet of HH, which they describe as “a facet associated with morality, integrity, and a reluctance to exploit others” (p. 21), three decidedly un-Machiavellian characteristics, was a particularly good predictor of favorable attitudes toward religion. These findings suggest that a dark mindset may be compatible with a rejection of religious precepts and principles. To be sure, further research is required to untangle the ostensibly complex nature of the relationship between Machiavellianism and social conservatism, broadly conceived. 

In considering this issue, it should be kept in mind that an important aspect of Machiavellianism involves contempt for conventional morality, which is seen as a senseless impediment to the pursuit of one’s self-interest. Machiavellians may be repelled by social conservatism insofar as that social philosophy represents a staunch defense of conventional values and moral positions. Similarly, it may be the case that religious social conservatives are viewed by Machiavellians as the public face of the traditional values they disdain. The extreme self-centeredness of people with high levels of this dark trait may create resentment toward any group that tries to put constraints on the attitudes and behaviors that define Machiavellianism, which is generally what conventional, established religions endeavor to do. If so, the name of this trait is appropriate, given that Machiavelli himself had many misgivings about Christianity’s influence on political leaders and humanity in general, and he made a number of statements in his books that have been interpreted as subtle digs against ecclesiastical officials. Apparently, the feeling was mutual—the church placed all of his works on their *Index of Forbidden Books*. Again, more research is needed to examine trait Machiavellianism’s association with socio-religious conservatism and its implications for political attitudes and behaviors.

It is also noteworthy that, as mentioned, a sizeable beta weight for Agreeableness in predicting socio-religious conservatism was reduced and rendered nonsignificant by the inclusion of the dark traits in the regressions. A meta-analytic review of the association between the Big Five personality factors and religion [85] found that of the five factors, Agreeableness was the strongest single predictor of religiosity. Further research that compares the impact of Machiavellianism versus Agreeableness and HH on socio-religious attitudes is clearly called for.

With regard to economic conservatism, the finding of no association between the Dark Triad and support for greater economic equality is not consistent with the theoretical perspective adopted here. This is somewhat surprising, given Bardeen and Michel’s [13] finding, noted earlier, that participants who had high levels of social liberalism and economic conservatism showed the highest levels of Machiavellianism. 

The theoretical position explored here suggested that a lower correlation should be found between the Dark Triad and overall political orientations compared to that between the dark traits and separate measures of social and economic liberalism–conservatism. There was some support for this position, in that the association between Machiavellianism and socio-religious conservatism was greater than that of Machiavellianism and overall liberalism–conservatism, although rather than being attributable to opposite-sign associations, it appears to have resulted from a robust negative association with socio-religious conservatism and no association with support for greater economic equality. However, this pattern was not observed for the other two dark traits. Nonetheless, the findings reported here underscore the importance of multi-dimensional measures of political orientation in future research on this topic, since it appears that single-item measures conflate the economic and social aspects of political orientation, which presents measurement problems insofar as the dark traits are differentially correlated with each dimension. 

At the zero-order level, Machiavellianism was a significant negative predictor of interest in politics, which is consistent with the theoretical position taken here, derived from the “spheres of control” perspective proposed by Paulhus [54], in which Machiavellians believe they have little control over sociopolitical matters. This finding is also consistent with a negative association between Machiavellianism and a propensity to vote, reported by Chen et al. [69]. However, although the coefficient remained negative in the regression analysis (β = −0.10), it failed to reach significance (*p* = 0.074). Further research into the association between an interest in politics and the Dark Triad would be beneficial. A study that includes a direct measure of perceived spheres of control, interest in politics, and all three dark traits would be helpful in that regard. 

## 7. Conclusions and Limitations

This study provided a theoretical perspective on how the Dark Triad relates to political orientations and interest in politics, using that perspective as a backdrop for an empirical analysis of those relationships. It also took steps to address some methodological issues found in earlier studies related to the use of abbreviated personality scales, the use of unidimensional measures of political orientations, and the absence of controls for the Big Five.

The theoretical approach advanced here bore some fruit, in that the association between Machiavellianism and socio-religious conservatism was consistent with the theoretical stance taken. The theoretical standpoint taken here was also shown to be consistent with findings from other studies, albeit ones that were not designed with this theoretical approach in mind. However, the lack of generalization to the other dark traits and a finding of no association between economic liberalism and the dark traits suggest that further development of this perspective is warranted. It is hoped that this study motivates researchers in this area to produce further theoretical work, especially with regard to how the prevalence of the Dark Triad in large groups affects political outcomes.

This study’s limitations include the fact that the sample was predominantly female, although sex was controlled for in the regressions, and when sex was introduced as a possible moderator, no significant moderating effects were observed. The sample was also bi-national, being composed of Canadians and Americans, with nationality controlled for in the regressions. Although a comparison of correlations reported here with those found in meta-analyses indicated that our sample was comparable to others used in this area of research, the use of more gender-balanced and single-nationality samples in future research would be beneficial. Finally, the participants in this research were not selected randomly, which places important limitations on the study’s external validity. Sometimes, in research of the sort done here, a choice must be made between using abbreviated measures with large, representative samples, or using full measures with smaller, non-representative samples. The latter strategy was pursued here, with its attendant strengths and weaknesses. 

## Figures and Tables

**Table 1:** Correlations between the Dark Triad, age, sex, and three political variables.

	Narcissism	Psychopathy	Machiavellianism	Age	Sex ^i^
**Political measure**					
Interest in politics	0.02	−0.07	−0.14 *	0.29 *	−0.15 *
Overall liberal–conservative orientation	−0.14 *	−0.02	−0.08	0.19 *	−0.09
Socio-religious conservatism Support for greater economic equality	−0.17 * 0.08	−0.12 * 0.07	−0.30 * 0.08	0.23 * 0.03	−0.03 −0.09
Mean	0.38	2.02	2.48		
Standard Deviation	0.17	0.36	0.38		

^i^ 1 = men; 2 = women; * *p* < 0.01, two-tailed.

**Table 2:** Correlations between the Big Five Personality factors and three political variables.

	Openness	Conscientiousness	Extraversion	Agreeableness	Neuroticism
**Political measure**					
Interest in politics	0.26 *	0.20 *	0.10	0.14 *	−0.20 *
Overall liberal–conservative orientation	−0.37 *	0.10	−0.05	0.03	−0.09
Socio-religious conservatism Support for greater economic equality	−0.36 * −0.24 *	0.07 0.09	−0.09 0.10	0.18 * −0.09	−0.04 −0.08
Mean Standard Deviation	158.30 19.10	171.78 19.20	163.36 19.19	175.53 17.16	136.76 23.41

* *p* < 0.01, two-tailed.

**Table 3:** Correlations between the Big Five Personality scales, the Dark Triad, age, and sex.

	Narcissism	Psychopathy	Machiavellianism	Age	Sex ^i^
**Personality scale**					
Openness	0.22 *	−0.05	−0.02	−0.23 *	0.17 *
Conscientiousness	0.02	−0.31 *	−0.36 *	0.15 *	0.02
Extraversion	0.35 *	−0.14 *	−0.01	−0.14 *	0.14 *
Agreeableness	−0.32 *	−0.46 *	−0.60 *	0.33 *	0.17 *
Neuroticism	−0.06	0.32 *	0.20 *	−0.31 *	0.13 *

^i^ 1 = men; 2 = women; * *p* < 0.01, two-tailed.

**Table 4:** Pearson’s *r* correlations * between the Big Five personality factors and overall political orientation ^i^.

	Openness	Conscientiousness	Extraversion	Agreeableness	Neuroticism
Current study	−0.37	0.10	−0.05	0.03	−0.09
Sibley et al. [73] meta-analysis, *all 73 studies*	−0.18	0.10	−0.01	−0.02	−0.03
Sibley et al. [73] meta-analysis, the *5 Canadian samples only*	−0.19	0.06	−0.08	0.04	0.06

* The correlations in the meta-analysis were transformed by Sibley et al. [73] to z-scores, weighted by inverse variance, and averaged prior to conversion to *r*-equivalent effect sizes.; ^i^ Higher scores indicate greater conservatism.

**Table 5:** Pearson’s *r* correlations between the Big Five personality traits and the Dark Triad observed in present study compared with meta-analyses conduced by Muris et al. [26] ^i^ and O’Boyle et al. [81].

	Narcissism	Psychopathy	Machiavellianism
**Big Five personality scale**			
*Openness*			
Current study	0.22	−0.05	−0.02
Muris et al. [26]	0.17	0.05	−0.00
O’Boyle et al. [81]	0.20	0.04	−0.04
***Conscientiousness***			
Current study	0.02	−0.31	−0.36
Muris et al. [26]	0.01	−0.27	−0.27
O’Boyle et al. [81]	0.09	−0.31	−0.21
***Extraversion***			
Current study	0.35	−0.14	−0.01
Muris et al. [26]	0.33	0.07	−0.10
O’Boyle et al. [81]	0.40	0.04	−0.01
***Agreeableness***			
Current study	−0.32	−0.46	−0.60
Muris et al. [26]	−0.29	−0.49	−0.51
O’Boyle et al. [81]	−0.29	−0.42	−0.39
***Neuroticism***			
Current study	−0.06	0.32	0.20
Muris et al. [26]	−0.06	−0.08	0.14
O’Boyle et al. [81]	−0.16	0.05	0.09

^i^ The correlations shown are not corrected for the shared variance among the three Dark traits. The Muris et al. [26] correlations are Fisher’s *z*-transformed coefficients, with effect sizes pooled across studies. O’Boyle et al. [81] use Hunter and Schmidt’s [82] equations to calculate the mean effect sizes; the figures shown are weighted mean correlations.

**Table 6:** Hierarchical Regression Analysis of Predictors of four Political Variables ^i^.

	Interest in Politics			Overall Liberal–Conservative Orientation			Socio-Religious Conservatism			Support for Greater Economic Equality		
	*Model 1*	*Model 2*	*Model 3*	*Model 1*	*Model 2*	*Model 3*	*Model 1*	*Model 2*	*Model 3*	*Model 1*	*Model 2*	*Model 3*
***Predictor variables***												
Sex ^ii^	−0.14 *	−0.23 ***	−0.23 ***	−0.10	−0.04	−0.03	−0.01	−0.01	0.01	−0.09	−0.03	−0.02
Age	0.25 ***	0.30 ***	0.31 ***	0.14 **	0.08	0.07	0.21 ***	0.11 *	0.11 *	0.03	0.03	0.03
Nationality ^iii^	0.14 *	0.16 *	0.14 *	0.22 ***	0.19 ***	0.18 ***	0.23 ***	0.21 ***	0.18 ***	0.10	0.08	0.09
Neuroticism		0.03	0.04		0.05	0.06		0.15 *	0.17 *		−0.01	−0.03
Extraversion		0.04	0.05		0.06	0.09		0.05	0.05		0.17 **	0.16 *
Openness		0.37 ***	0.37 ***		−0.35 ***	−0.34 ***		−0.35 ***	−0.35 ***		−0.28 ***	−0.28 ***
Agreeableness		0.05	0.00		−0.01	−0.07		0.20 **	0.11		−0.13 *	−0.09
Conscientiousness		0.09	0.08		0.11	0.11		0.05	0.03		0.06	0.06
Narcissism			−0.03			−0.15 *			−0.09			0.03
Machiavellianism			−0.10			−0.12 *			−0.29 ***			0.12
Psychopathy			0.02			0.08			0.09			−0.04
*R ^2^*	0.11 ***	0.25 ***	0.25 ***	0.08 ***	0.19 ***	0.18 ***	0.10 ***	0.24 ***	0.30 ***	0.02	0.10 ***	0.11 ***
∆*R ^2^*		0.16 ***	0.01		0.11 ***	0.02 *		0.14 ***	0.06 ***		0.09 ***	0.01

^i^ Standardized Regression Coefficients. ^ii^ 1 = men; 2 = women; ^iii^ 1 = Canadian; 2 = American. * *p* < 0.05; ** *p* < 0.01; *** *p* < 0.001.

## Data Availability

Data can be made available by contacting the first, third, or fourth authors.
